# Novel Tear Biomarkers in Ocular Graft Versus Host Disease Associated with Th1/Th2 Immune Responses: A Case Series and Literature Review

**DOI:** 10.3390/ijms26094311

**Published:** 2025-05-01

**Authors:** Mihaela-Madalina Timofte-Zorila, Mariana Pavel-Tanasa, Daniela Constantinescu, Corina Cianga, Daniel Constantin Branisteanu, Giuseppe Giannaccare, Filippo Lixi, Angela Dascalescu, Nicoleta Vlas, Sabina Turcas, Cristina Preda

**Affiliations:** 1 Faculty of Medicine, Grigore T. Popa University of Medicine and Pharmacy, 700115 Iasi, Romania; mihaela-madalina.timofte-zorila@d.umfiasi.ro (M.-M.T.-Z.); daniel.branisteanu@umfiasi.ro (D.C.B.); vlas.nicoleta@email.umfiasi.ro (N.V.); turcas.sabina@email.umfiasi.ro (S.T.); 2 Department of Ophthalmology, Cai Ferate Clinical Hospital, 700506 Iasi, Romania; 3 Department of Immunology, Grigore T. Popa University of Medicine and Pharmacy, 700115 Iasi, Romania; d.constantinescu@umfiasi.ro (D.C.); corina.cianga@umfiasi.ro (C.C.); 4 Laboratory of Immunology, St. Spiridon County Clinical Emergency Hospital, 700111 Iasi, Romania; 5 Eye Clinic, Department of Surgical Sciences, University of Cagliari, 09124 Cagliari, Italy; giuseppe.giannaccare@unica.it (G.G.); f.lixi1@studenti.unica.it (F.L.); 6 Department of Hematology, Grigore T. Popa University of Medicine and Pharmacy, 700115 Iasi, Romania; angela.dascalescu@umfiasi.ro; 7 Endocrinology Department, Grigore T. Popa University of Medicine and Pharmacy, 700115 Iasi, Romania; cristina.preda@umfiasi.ro

**Keywords:** ocular GVHD, CD27, Fas, TRAIL, TRAIL-R2, cytokines, eye inflammation

## Abstract

Ocular graft versus host disease (oGVHD) is a common complication of allogeneic hematopoietic stem cell transplantation and may be associated with dry eye disease and chronic inflammation and fibrosis. Immune dysregulation, particularly the Th1/Th2 imbalance, plays a key role in the progression of oGVHD. This case study presents two oGVHD patients (a 20-year-old with acute oGVHD and a 59-year-old with chronic oGVHD), analyzing clinical dry eye parameters (Schirmer test I, tear film break-up time, Ocular Surface Disease Index (OSDI), and kerato-conjunctival staining) alongside tear biomarkers. A 27-plex tear cytokine analysis was performed using the Luminex200 platform, assessing various biomarkers against a control group-defined normal range. Key biomarkers included beta2-microglobulin (β2-MG), complement components, chemokines, growth factors, and both pro-inflammatory and anti-inflammatory cytokines, as well a series of soluble ligand and receptors. The study identified distinct biomarker progression patterns during topical corticosteroid treatment in the acute oGHVD patient, suggesting potential shifts in Th1/Th2 responses as the disease progressed. Notably, the soluble CD27, TNF-related apoptosis-inducing ligand (TRAIL) receptor 2 (TRAIL-R2), chemokine ligand 2 (CCL2), and IL-1β, initially elevated, normalized during treatment, while tear-soluble Fas remained highly elevated (>400-fold). Conversely, soluble TRAIL, which was initially at very low levels (100-fold lower), increased during treatment and reached normal tear levels, coinciding with improvements in the clinical ocular inflammation symptoms and OSDI score. This case study also highlights potential differences between acute and chronic oGVHD, particularly in the distinct patterns of novel tear biomarkers such as CD27, TRAIL/TRAIL-R2, and CCL2. Enhancing our understanding of biomarker dynamics may improve disease monitoring and pave the way for personalized management strategies to improve patient outcomes.

## 1. Introduction

Graft versus host disease (GVHD) is a systemic illness characterized by an increased interplay of immunological-mediated reactions triggered by donor immune cells that recognize recipient antigens [1]. Since allogenic hematopoietic stem cell transplantation (allo-HSCT) is increasingly used to treat various hematologic conditions, patients often develop GVHD despite receiving immune prophylaxis therapy. This complication affects up to 70% of patients and is the leading cause of late mortality following the procedure [2]. GVHD is typified by a dysregulated immunological response in which host tissues are targeted by donor immune cells, specifically T cells. The recognition of host antigens by donor T cells causes acute GVHD, while chronic GVHD is characterized by immune cell infiltration and an inappropriate immunological response by allo- and auto-reactive T cells [3]. The donor T cells play a central role in the altered immunoinflammatory response observed in GVHD. Following interactions with host and donor antigen-presenting cells (APCs) and engagement with major histocompatibility complex (MHC) molecules—class I (CD8+ T cells) and class II (CD4+ T cells)—the donor T cells become activated and differentiate into effector CD8+ and CD4+ cells. This activation initially drives a Th1 response, characterized by the production of pro-inflammatory cytokines, including interferon-gamma (IFN-γ), interleukin-2 (IL-2), and tumor necrosis factor-alpha (TNF-α), which mediate tissue damage and destruction [3,4]. While the pathophysiology of acute GVHD is relatively well understood, the mechanisms underlying chronic GVHD still remain unclear. Nonetheless, effector T cells, Tregs, and B cells, along with their associated cytokines, are believed to play a central role in the disease’s pathogenesis [3].

Ocular GVHD (oGVHD) mainly impacts the ocular surface, with conjunctival or corneal epithelial alterations, dysfunction of the lacrimal and meibomian glands, eyelid margin abnormalities, and dry eye disease (DED) presenting as keratoconjunctivitis sicca (KCS) [5,6]. Donor-derived immune cells, including T lymphocytes, macrophages, and neutrophils, traverse the vascular barrier into ocular tissues, perpetuating a localized inflammatory cascade that contributes to progressive alteration of the ocular surface and adnexa [7]. Ocular manifestations of GVHD are predominantly characterized by DED, though the condition may present with a spectrum of clinical symptoms [8]. The anterior segment involvement progresses over time, affecting 19.7% of patients at one year post-allo-HSCT, increasing to 29.3% at two years, 40.7% at three years, 47.2% at four years, and reaching 49.7% at five years [9]. These data underscore the progressive nature of ocular GVHD (oGVHD) and its significant impact on the anterior segment over time.

Currently, the diagnosis of ocular GVHD primarily relies on clinical evaluation and symptom assessment, which often lack specificity for the condition. Advances in understanding the pathophysiology of GVHD have elucidated mechanisms beyond donor–host cell interactions, highlighting the role of sustained immune activation triggered by tissue damage and cytokine release [8]. In oGVHD, ocular tissue damage initiates a feedback loop where recruited immune cells amplify cytokine and chemokine production, perpetuating inflammation and further tissue destruction. Tear fluid analyses have consistently demonstrated elevated levels of cytokines (e.g., IFN-γ, IL-6, IL-8, IL-10, IL-12, IL-17A, MMP-9, neutrophil elastase, and VEGF) and chemokines (CXCL8 and CXCL10) [4]. The pursuit of reliable diagnostic or prognostic biomarkers remains ongoing, with tear proteomics emerging as a promising avenue due to the feasibility of noninvasive, repetitive sample collection and its potential to provide insights into disease progression [10,11].

In this study, we present a unique panel of tear biomarkers designed to monitor the progression of inflammation in a patient with acute ocular GVHD over the course of one month, in comparison to a chronic case and a healthy control group involving 13 healthy individuals. The potential applications of this biomarker panel include facilitating early diagnosis of the disease, enhancing our understanding of its immunological regulation, and assessing clinical responses to treatment options. Furthermore, this panel proposes preliminary insights into novel biomarkers to be used in further larger cohort studies for discriminating between acute and chronic oGVHD, and investigating the underlying pathological mechanisms of ocular disorders associated with GVHD progression.

## 2. Results

### 2.1. Case Presentation

**Case 1—Acute GVHD.** A 20-year-old male patient with a history of acute myeloblastic leukemia underwent an allo-HSCT from a haploidentical donor (father) using peripheral blood stem cells (PBSC). Before stem cell transplantation, the patient was not on any systemic medication known to cause or contribute to dry eye disease. On day 109 post-transplant, he developed acute GVHD following the cessation of immunosuppressive therapy on day 90. The GVHD initially presented as grade II cutaneous involvement, for which intravenous Methylprednisolone was administered at a dose of 250 mg/day in combination with dexamethasone (16 mg/day). Despite initial therapy, the condition progressed to grade IV GVHD, with subsequent involvement of the oral mucosa and ocular complications.

The patient had no prior history of DED, nor had undergone any previous ophthalmological evaluation. Before referring the patient for an ophthalmological examination, the hematologist initiated systemic corticosteroid therapy and prescribed topical eye drops containing tobramycin and dexamethasone.

**Figure 1:**
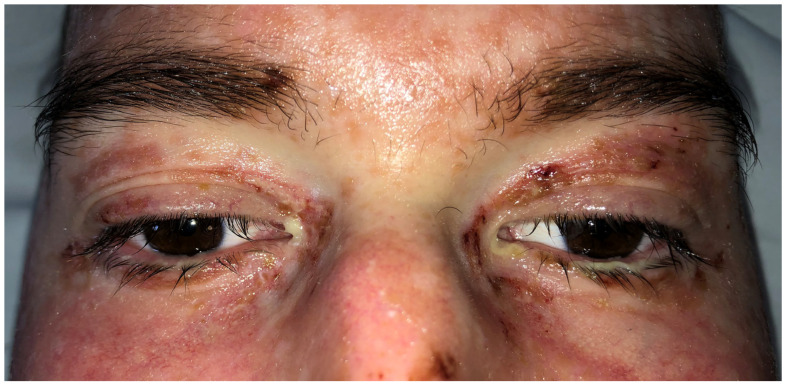
Case 1: clinical ophthalmological examination at T0 (7 days after symptoms’ onset).

An initial ophthalmological examination was performed at the patient’s bedside after seven days of persistent symptoms (T0, Figure 1). The Ocular Surface Disease Index (OSDI) score was 70, indicating severe ocular surface disease. The anterior segment assessment revealed muco-filamentous secretions agglutinating the eyelid skin, erythema with excoriation, and hemorrhagic crusting of the lid margins, which caused significant discomfort and impeded the examination. Mild conjunctival hyperemia and papillary conjunctivitis were observed, while the cornea was clear without areas of fluorescein staining. The anterior chamber and the lens were normal. Fundus examination revealed no abnormalities. The Schirmer type I test (without anesthetic) measured 30 mm for the right eye (RE) and 25 mm for the left eye (LE), and the Schirmer strips were immediately frozen at −80 °C until further analysis. The tear film break-up time (TBUT) was 5 s in the RE and 6 s in the LE. Swabs were taken from both eyes for bacterial culture, but the results were negative, likely due to the use of topical antibiotics for 5 days. The patient was treated on both eyes with a combination of chloramphenicol and dexamethasone eye drops four times daily, along with nocturnal ointment with the same active principle, for 10 days. Preservative-free artificial tears were administered four times daily, and lid hygiene was prescribed as part of the therapeutical management. After T0, Methylprednisolone was increased to 500 mg/day for 3 days, then dropped to 125 mg/day when 10 mg twice daily Ruxolitinib and Tacrolimus (in variable doses) were added.

**Figure 2:**
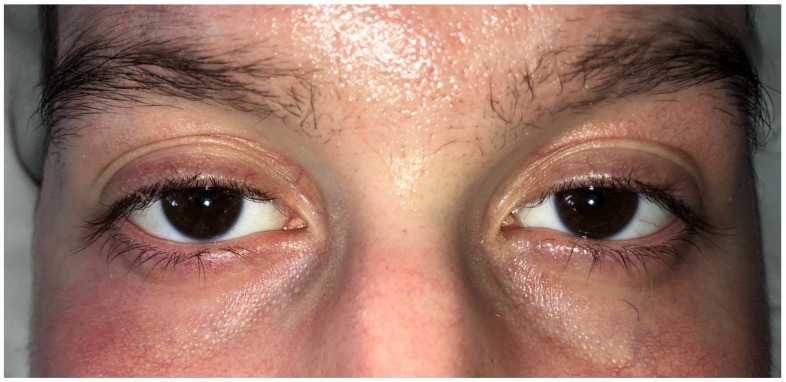
Case 1: clinical ophthalmological examination at T1 (10 days after T0).

After 10 days (T1), a follow-up ophthalmologic examination revealed resolution of the ocular discharge and redness, with the eyelids returning to a normal aspect (Figure 2). However, mild stenosis of the lacrimal puncta was noted in both eyes, along with meibomian gland dysfunction. The OSDI score was 30, indicating a moderate degree of ocular surface disease. Tear samples were collected using Schirmer strips, as previously described. The Schirmer type I test measured 7 mm for the RE and 8 mm for the LE, while TBUT measured 6 s in both eyes. The recommended treatment included Flumetholon eye drops administered three times daily in both eyes for the subsequent 10 days, continuing the regimen of preservative-free artificial tears and lid hygiene. Ruxolitinib was administered at a dose of 10 mg twice daily, in combination with 80 mg of Methylprednisolone, dexamethasone 16 mg/day, and Tacrolimus 2 mg/day until the cutaneous lesions were completely resolved. Following that, Methylprednisolone was lowered to 20 mg/day and dexamethasone was stopped before the third ophthalmological control.

**Figure 3:**
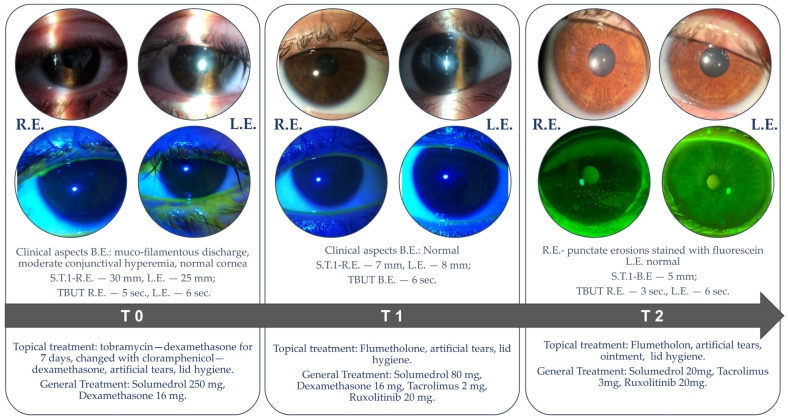
Case 1: Slit-lamp examination at different time points. T0—first ophthalmological evaluation performed after 7 days of persistent symptoms (treated with tobramycin—dexamethasone); T1—10 days after T0; T2—10 days after T1. R.E. = right eye, L.E. = left eye, B.E. = both eyes, S.T.1 = Schirmer test type I, TBUT = tear break-up time.

Twenty days after the first ophthalmologic assessment (T2), the Best-Corrected Visual Acuity (BCVA) was 1.0 in both eyes, and the intraocular pressure was within normal limits. The OSDI score was 34, reflecting a significant reduction in symptoms compared to T0. The RE presented inferior corneal fluorescein staining (Oxford score 2), while the LE appeared normal. The Schirmer type I test results measured 5 mm for both eyes, while the TBUT was 3 s in the RE and 6 s in the LE (Figure 3).

Tear samples were collected and immediately frozen as previously mentioned. Local treatment with Flumetholon eye drops was tapered by reducing one drop every five days, and then discontinued after 10 days. Ointments at bedtime were introduced, while continuing the use of preservative-free artificial tears and adhering to lid hygiene. The final diagnosis was bilateral keratoconjunctivitis sicca in the context of oGVHD, associated with tear film instability in the RE. Systemic therapy with Ruxolitinib was lowered to 5 mg twice daily and Tacrolimus was maintained for 14 days after the last ophthalmological evaluation. Ruxolitinib was discontinued due to severe secondary cytopenia and the complete resolution of GVHD-related symptoms. The patient continued treatment with Tacrolimus for two months following the final ophthalmological check-up.

**Case 2—Chronic GVHD.** A 59-year-old male with a history of acute myeloblastic leukemia with FLT3 internal tandem duplication (AML-FLT3/ITD) underwent an allogeneic stem cell transplant using PBSC from a 100% HLA-matched related donor (sister) in August 2023. Before stem cell transplantation, the patient was not on any systemic medication known to cause or contribute to dry eye disease. On day 141 post-transplant he developed GVHD with hepatotoxicity and pulmonary complications. On day 227 post-transplant (March 2024), the patient presented with symptoms of cutaneous GVHD, characterized by grade I–II lesions, for which he received topical corticosteroid treatment and systemic corticosteroid therapy with Methylprednisolone. This treatment was continued until May 2024. Following this period, he did not require systemic immunosuppressive therapy.

In August 2024, he presented to the Ophthalmology Department with complaints of conjunctival congestion, symptoms of dry eyes, and blurred vision in the left eye. Notably, the patient had no prior history of DED, neither had undergone previous ophthalmologic evaluation.

**Figure 4:**
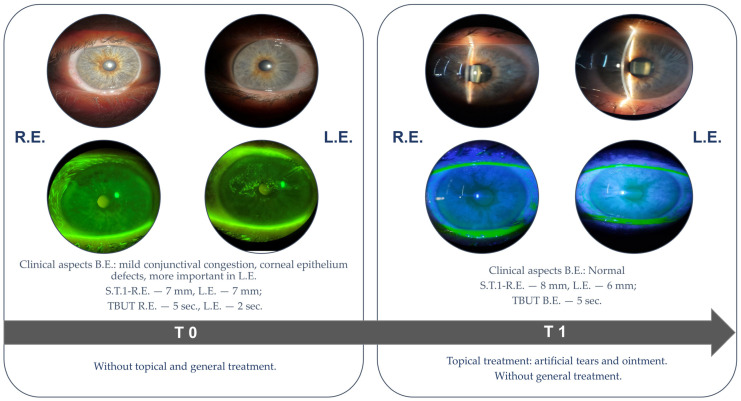
Case 2: Slit-lamp examination at different time points. T0—first ophthalmological evaluation; T1—10 days after T0. R.E. = right eye, L.E. = left eye, B.E. = both eyes, S.T.1 = Schirmer test type I, TBUT = tear break-up time.

The initial ophthalmic examination (T0) revealed a BCVA of 1.0 in the RE and 0.9 in the LE, while the intraocular pressure was within normal limits. The OSDI score was 32, indicating a moderate degree of ocular surface disease. The Schirmer type I test measured 7 mm in both eyes, and the TBUT was 5 s in the RE and 2 s in the LE. Slit-lamp examination revealed mild conjunctival congestion and punctate epithelial fluorescein staining of the cornea, more pronounced in the LE. The kerato-conjunctival staining, according to the Oxford grading scale, was grade 1 in the RE and grade 3 in the LE (Figure 4). Tear samples were obtained using the Schirmer’s type I test and preserved as described above before further analysis.

The patient was prescribed preservative-free artificial tears containing a high concentration of hyaluronic acid to be administered three times daily, along with a vitamin A-based ophthalmic ointment for use at night. At 10 days follow-up (T1, Figure 4) the patient presented significant improvement in the ocular surface (without corneal staining) and effective control of dry eye symptoms (OSDI score was 11). The use of lubricant tears and regular ophthalmologic evaluations were recommended to monitor potential ocular complications associated with cGVHD.

**Control Group.** The control group involved 13 healthy volunteers (mean age of 29 ± 5 years old) who were first clinically examined to ensure their normal ocular status. All controls fulfilled the inclusion/exclusion criteria.

### 2.2. Tear Analysis

Tear film samples were collected at various stages of disease progression from the acute case of oGVHD, alongside the chronic oGVHD case and healthy control subjects, to define the individual profiles of tear-soluble biomarkers (Table 1).

**Figure 5:**
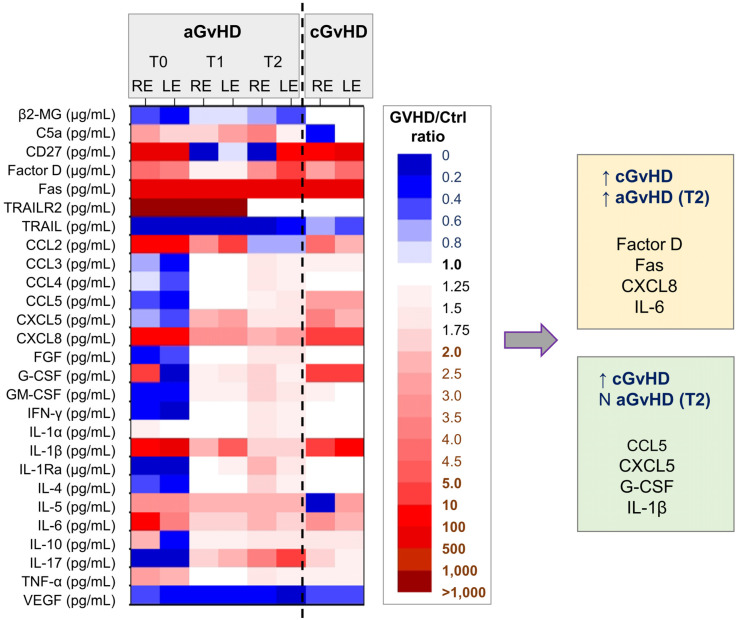
Heat map of soluble biomarker profiles in tears collected from GVHD cases: (**left**) acute GVHD at different time points (T0, T1, and T2), (**right**) chronic GVHD case. Blue = the value was smaller than in controls, red = the value was larger than in controls. The factors increased in both cases included the following: factor D, Fas, CXCL8, and IL-6. The following factors increased only in the chronic case: CCL5, CXCL5, G-CSF, and IL-1β. N = normal values; ↑ = increased values.

Representing each value of the GVHD either as lower, within the range, or exceeding the control values (defined by the minimum and maximum limits), the tear-soluble biomarkers showed distinct patterns of evolution over time: decreasing, increasing, or showing only moderate changes. These patterns occasionally differed between the right and left eye, as expected given the disparity in the clinical severity of ocular disease between the two eyes (Figure 1). When comparing the biomarkers’ profile at T2 (20 days after the initial ophthalmological evaluation and 27 days since symptoms’ onset), a group of elevated biomarkers was observed in both cases (Factor D [12], Fas/CD95 [13,14,15], TRAIL-R2/DR5/TNFRSF10B [16,17,18], CXCL5, and IL-6), while another group of biomarkers was specifically heightened in the chronic GVHD case (Figure 5—right side: CCL5, CXCL5, G-CSF, and IL-1β).

**Figure 6:**
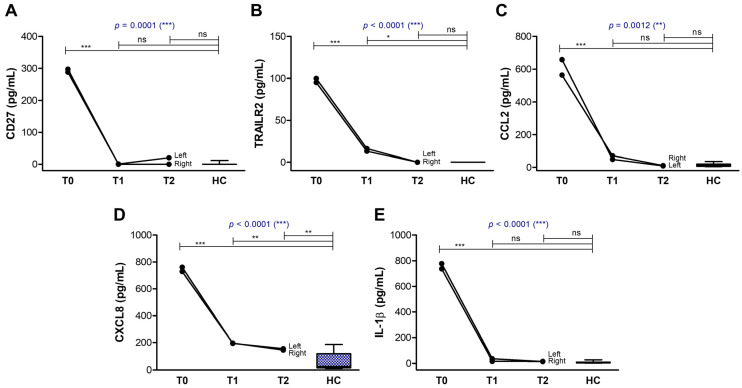
Graphic representation of biomarkers showing a decline over time, similar in both eyes. The lines indicate the progression of biomarkers in both eyes (right and left): (**A**) CD27; (**B**) TRAILR2; (**C**) CCL2; (**D**) CXCL8; (**E**) IL-1β. Controls are indicated as box plots with min and max values (*** *p* < 0.001, ** *p* < 0.01, * *p* < 0.05, ns—not significant; two-way repeated measures ANOVA with Bonferroni Multiple Comparison test).

**Figure 7:**
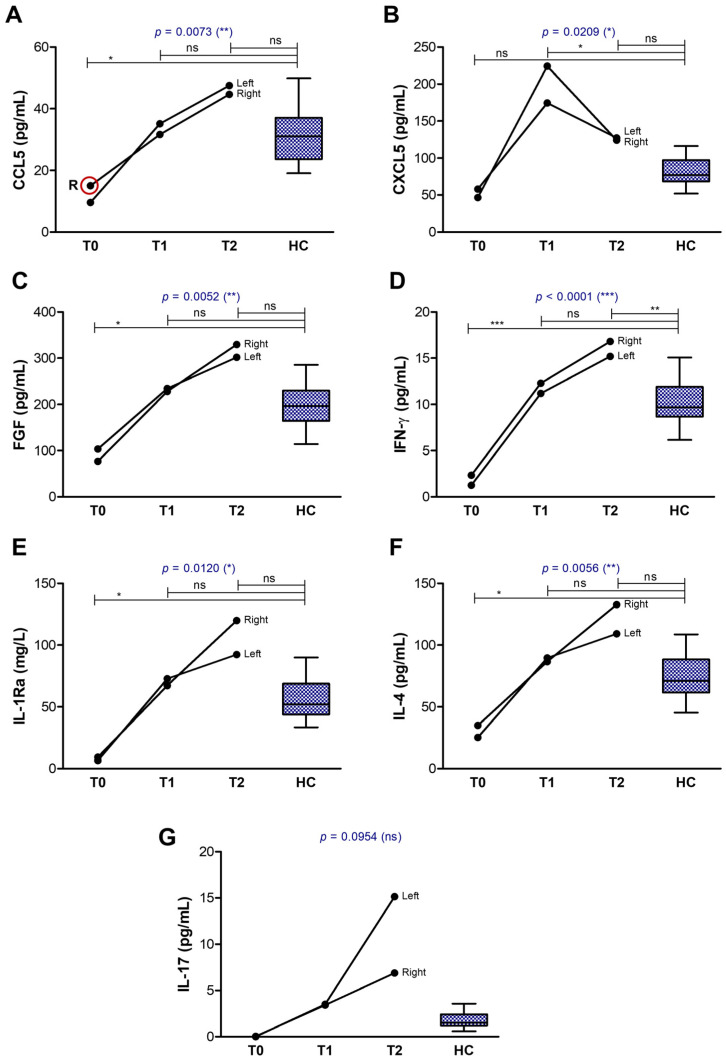
Graphical representation of biomarkers showing an increase over time, similar in both eyes. The lines indicate the progression of biomarkers in both eyes (right and left): (**A**) CCL5; (**B**) CXCL5; (**C**) FGF; (**D**) IFN-γ; (**E**) IL-1Ra; (**F**) IL-4; (**G**) IL-17. Controls are indicated as box plots with min and max values (*** *p* < 0.001, ** *p* < 0.01, * *p* < 0.05, ns—not significant; two-way repeated measures ANOVA with Bonferroni Multiple Comparison test). R indicates the initial value in the right eye, while the red circle highlights cases where the initial value in the right eye was more than 50% higher than that in the left eye.

The biomarkers with extremely high initial values in the tear film collected at T0, that showed a clear decline over time until reaching the normal range of values, included the soluble form of the co-stimulatory molecule CD27, the soluble receptor TRAIL-R2, the pro-inflammatory chemokines CCL2/MCP-1 and CXCL8, and the pro-inflammatory cytokine IL-1β (Figure 6). A second distinct group of biomarkers was characterized by initially very low levels at T0, which normalized or even increased above the control range at T2, and included the anti-inflammatory chemokines CCL5 and CXCL5, and the FGF growth factor involved in tissue repair which exhibits strong anti-inflammatory effects, further potentiated by the soluble interleukin-1 receptor antagonist (IL-1Ra) (Figure 7). Since the induction of IL-1Ra is promoted by the anti-inflammatory mechanisms mediated by IL-4 and IFN-γ (an immune cytokine with dual roles) concomitant with IL-1β reduction [19,20], IL-1Ra, IL-4, and IFN-γ tear levels showed a similar increasing trend accompanied by a simultaneous decrease in IL-1β levels in our aGVHD case (Figure 6E and Figure 7D–F). The initially high IL-1β values (over 700 pg/mL, compared to control values of 7.74 ± 1.89 pg/mL) were not accompanied by IL-1α, and only moderately by TNF-α (Table 1).

**Figure 8:**
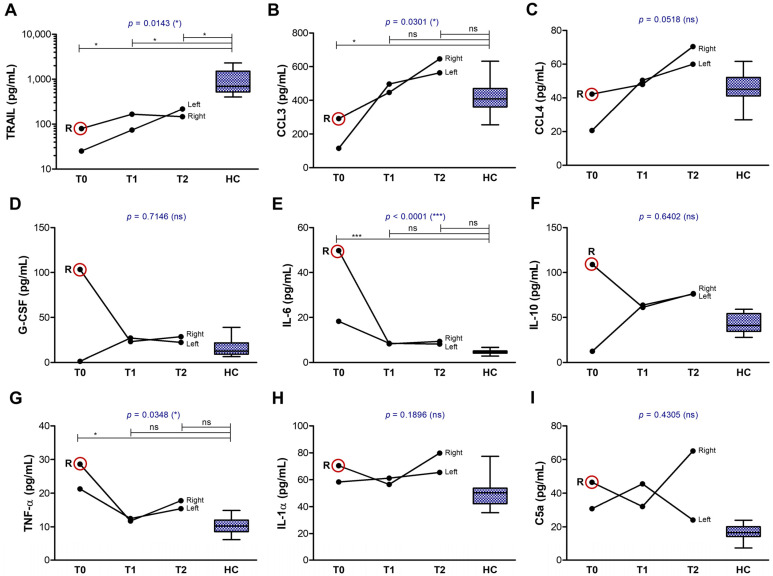
Graphical representation of biomarkers showing pattern differences between the right and left eyes. The lines indicate the progression of biomarkers in both eyes (right and left): (**A**) TRAIL; (**B**) CCL3; (**C**) CCL4; (**D**) G-CSF; (**E**) IL-6; (**F**) IL-10; (**G**) TNF-α; (**H**) IL-1α; (**I**) C5a. Controls are indicated as box plots with min and max values (*** *p* < 0.001, * *p* < 0.05, ns—not significant; two-way repeated measures ANOVA with Bonferroni Multiple Comparison test). R indicates the initial value in the right eye, while the red circle highlights cases where the initial value in the right eye was more than 50% higher than that in the left eye.

Another group of soluble factors showed at least 50% difference between the right and left eyes, with initial values being higher in the right eye (Figure 8A–I). This group included the soluble form of TRAIL, a membrane protein expressed specially by B and Th2 lymphocytes, which may be proteolytically cleaved by matrix metalloproteinases (MMPs) and a disintegrin and metalloproteinases (ADAMs) [21,22,23], which are released upon high levels of systemic inflammation (Figure 8A). For instance, MMP-1 and MMP-9 are secreted by macrophages when stimulated with the complement component C5a, released in high amounts by the systemic complement activation characterizing the GVHD [24,25,26]. The initial higher levels of soluble TRAIL in the right eye compared to the left eye was accompanied by and showed a similar pattern with CCL3 and CCL4 chemokines (Figure 8B,C), potentially secreted by activated B cells and professional antigen-presenting cells (APCs) [27,28]. G-CSF, which is known to enhance the immunoglobulin generation by activated B cells [29], was massively upregulated in the right eye at T0, similar to the Th2 cytokines IL-6 and IL-10, which are known to potentiate the proliferation and differentiation of activated B cells into plasma cells [30]. Intense stimulation with pro-inflammatory cytokines like IL-6 can abnormally activate certain subsets of B cells to become regulatory B cells that secrete IL-10 [31,32]. This process maintains elevated levels of this potent anti-inflammatory cytokine, which plays important roles in negatively regulating immune responses. Indeed, the IL-10 and IL-6 levels at T2 were elevated by at least 50% in both eyes compared to the normal range, while G-CSF levels normalized (Figure 8D–F).

**Figure 9:**
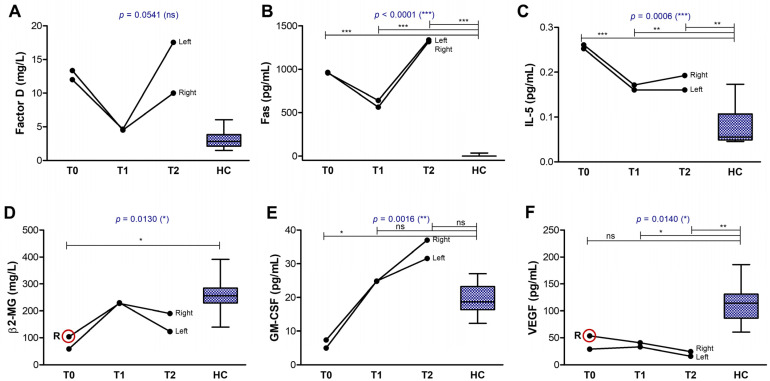
Graphical representation of biomarkers showing only a moderate overall change. The lines indicate the progression of biomarkers in both eyes (right and left): (**A**) factor D; (**B**) Fas; (**C**) IL-5; (**D**) β2-MG; (**E**) GM-CSF; (**F**) VEGF. Controls are indicated as box plots with min and max values (*** *p* < 0.001, ** *p* < 0.01, * *p* < 0.05, ns—not significant; two-way repeated measures ANOVA with Bonferroni Multiple Comparison test). R indicates the initial value in the right eye, while the red circle highlights cases where the initial value in the right eye was more than 50% higher than that in the left eye.

A few soluble factors maintained elevated levels at both T0 and T2 in the aGVHD case, as well as in the cGVHD case, such as the complement components C5a (Figure 8I) and Factor D (Figure 9A), and soluble membrane receptor Fas.

The Fas/FasL pathway is involved in the T cell-mediated GVHD, since FasL on T cells engage with Fas on target cells, inducing their apoptosis, and potentially the release of soluble Fas molecules [33,34,35]. As expected, the soluble Fas levels in the tear film reached values over 1000 pg/mL in both acute and chronic GVHD cases, much above the barely detectable levels in the controls (mean value of 2.69 pg/mL, Figure 9B). The last subgroup of molecules showed either decreased or normal values with moderate changes over time, including the β2-MG, GM-CSF, and VEGF in both cases of GVHD (Figure 9C–E).

**Figure 10:**
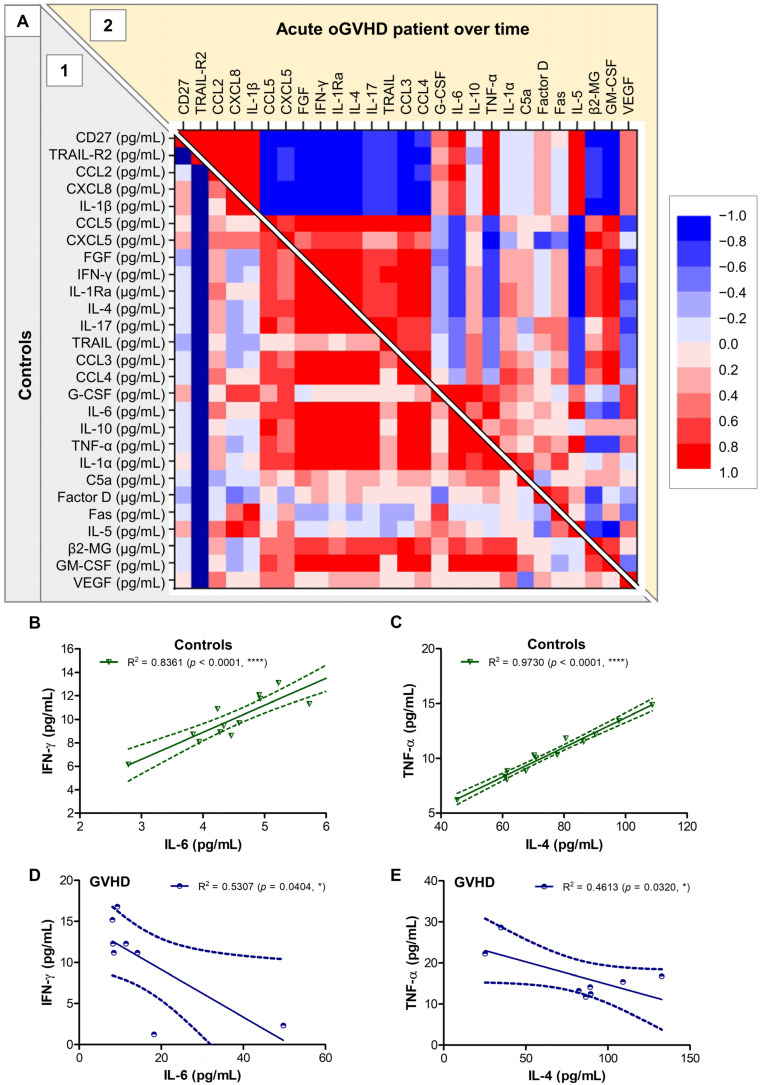
Regression statistics describing the associations of distinct soluble biomarkers in tears in the control group and the acute oGVHD patient monitored over time. (**A**) Heat map of correlation coefficients (R) for controls (1 = left-bottom) or the acute oGVHD case (2 = right-upper). Linear regression analysis for (**B**) IL-6 and IFN-γ or (**C**) IL-4 and TNF-α in controls, and for (**D**) IL-6 and IFN-γ or (**E**) IL-4 and TNF-α in the two oGVHD patients, represented as best-fit line with 95% confidence band (**** *p* < 0.0001, * *p* < 0.05; Pearson test).

The correlation analysis of various tear biomarkers in the control group, and in the acute case of GVHD monitored over time, allowed the visualization of several clusters of biomarkers. In control subjects, the group comprising IL-4, IL-5, IL-6, IL-10, IL-1Ra, TNF-α, IFN-γ, C5a, Factor D, Fas, β2-MG, GM-CSF, and CCL3 eventuated as a cluster of strong positive associations. Conversely, in the GVHD, two other clusters emerged: the cluster formed by IL-1β, IL-6, TNF-α, CD27, TRAIL-R2, CCL2, and CXCL8 negatively correlated with the cluster comprising IL-4, IL-1Ra, IL-17, FGF, β2-MG, GM-CSF, CCL3, CCL4, CCL5, and CXCL5 (Figure 10A). For instance, if IL-6 and IFN-γ or IL-4 and TNF-α showed strong positive associations within the control group (R^2^ = 0.84 and R^2^ = 0.97, respectively; *p* < 0.0001), these associations were not visible in the acute oGVHD patient monitored over time (R^2^ = 0.52 and R^2^ = 0.49, respectively) (Figure 10B–E). These data visualizations reinforced the previously identified clusters of progress patterns of biomarkers, suggesting an initial strong Th1 response that gradually diminished, being subsequently replaced by Th2 and/or Th17 responses over the treatment period with topical corticosteroids.

## 3. Discussion

Ocular GVHD is a significant complication of allo-HSCT, characterized by chronic inflammation of the ocular surface. The immune dysregulation underlying ocular GVHD involves an imbalance in CD4+ T cell responses, specifically between the Th1 and Th2 pathways. In acute ocular GVHD, the Th1 response is typically dominant, driven by the release of pro-inflammatory cytokines such as IFN-γ, IL-6, and TNF-α. This Th1-skewed immune environment contributes to tissue damage, disruption of the tear film, and meibomian gland dysfunction [3,4]. Conversely, a reduced Th2 response, which normally aids in maintaining immune tolerance through cytokines like interleukin-4 (IL-4) and interleukin-10 (IL-10) and facilitates appropriate B cell activation and function, can exacerbate the inflammatory process. Thus, acute GVHD is primarily characterized by a pro-inflammatory Th1 response, whereas chronic GVHD is predominantly associated with Th2-mediated inflammation [7,36]. Understanding the Th1/Th2 imbalance in oGVHD has important therapeutic implications, since interventions aimed at modulating this balance may help reduce inflammation and preserve ocular surface integrity. This process is further complicated by abnormal activation of the complement C3/CD4+ T cells’ axis, which impacts the corneal neurosensory layer, potentially causing neurotrophic ulcers [37,38].

Here, we presented two cases of ocular GVHD (one acute and one chronic) and examined the impact of treatment on the progression of tear biomarkers, while also defining the normal value range of these biomarkers within tear fluid in relation to disease progression. Our preliminary observations are limited to only two cases of oGVHD, being only hypothesis-generating findings, and extensive validation on larger cohorts is warranted in further studies.

### 3.1. Ophthalmological Examination Status

For the acute oGVHD patient, the initial Schirmer type I test revealed tear production of 30 mm in the RE and 25 mm in the LE, reflecting a high degree of ocular inflammation. As part of the natural course of the pathology, fibrosis of the lacrimal and meibomian glands progressively developed, leading to a reduction in tear production over time. Consequently, the Schirmer type I values decreased to 7 mm in the RE and 8 mm in the LE at T1, eventually declining to 5 mm in both eyes at T2. This suggests a decline in the overall function of the lacrimal system, which is typical in the progression of GVHD. Tear film stability was also assessed, using TBUT as a biomarker of ocular surface health. In the LE, TBUT remained stable at 6 s throughout the corticosteroid treatment, which is considered below the normal range (typically >10 s), indicating a mild to moderate dry eye disease. However, TBUT decreased significantly to 3 s in the RE at T2, suggesting a more severe progression of GVHD and further compromise of the ocular surface. Thus, we could conclude that for the acute oGVHD patient, the RE was characterized by an initially higher degree of inflammation, which showed a more severe progression towards dry eye disease when compared to the LE, despite identical local treatment for both eyes.

In the chronic case, the initial Schirmer type I test measured 7 mm in both eyes with differences in TBUT measurements: 5 s for the RE and 2 s for the LE. While the Schirmer test remained unchanged during treatment, TBUT improved to 5 s in the LE. In this patient, the left eye (LE) initially exhibited more severe dry eye disease than the RE, with a corneal staining score of three (moderate) in the LE versus one (minimal) in the RE, according to the Oxford grading scale. Following treatment, clinical improvement was observed in the LE. The Ocular Surface Disease Index (OSDI) was assessed at T0 and T1, demonstrating a reduction from 32 to 11, thus indicating a significant improvement in symptom severity.

These observed changes in the initial degree of inflammation and dry eye symptoms were also reflected in the differing initial levels of some tested biomarkers, as discussed below.

### 3.2. Tear Biomarkers

Particularly for these two patients, we were able to categorize the soluble markers investigated in tear fluid into three main groups: (i) those with extremely high initial values (around 100-fold the normal range) that decrease over time, (ii) those that increase over time (with changes related or not to clinical progression), and (iii) those that remain consistently elevated or decreased. Starting from these categories of biomarkers, we will next discuss the findings in the two investigated patients with oGVHD in relation to a comprehensive review of the data reported in the literature.

#### 3.2.1. The First Group of Tear Biomarkers

This group included the following molecules: the soluble form of the co-stimulatory molecule CD27, the soluble receptor TRAIL-R2, the pro-inflammatory chemokines CCL2/MCP-1 and CXCL8, and the pro-inflammatory cytokines IL-1β and TNF-α. The reduction of these biomarkers in tears suggests an initial Th1 response that significantly diminished over the course of the treatment. The levels of these cytokines correlated with the Schirmer type I test measurements, and their dynamics over time provided insight into disease progression, indicating a transition from high initial inflammation towards fibrosis.

CD27, a member of the tumor necrosis factor receptor superfamily, plays a critical role in modulating immune responses. Precisely, CD27 is implicated in the dysregulated T cell activity leading to chronic inflammation and tissue damage, and elevated CD27 signaling can exacerbate T cell activation and cytokine production, contributing to the breakdown of immune tolerance in various tissues [39]. Importantly, T cell overactivation is thought to perpetuate the fibrosis, lacrimal gland dysfunction, and ocular surface damage characteristic of GVHD [39]. The soluble CD27 may result from the proteolytical cleavage of its membrane-bound form on the T cell surface, a process carried out by metalloproteinases that are released in high amounts during the inflammatory process [40]. Importantly, in our study, high levels of soluble CD27 were also observed in the chronic oGVHD patient, this study being the first one, to our knowledge, to determine the values of this co-stimulatory molecule in tear fluid collected from GVHD patients.

Two other important ligand–receptor systems implicated in the regulation of Th1 and Th2 responses are the TRAIL/TRAIL-R1/2 and FasL/Fas systems. Both ligands (TRAIL and FasL) can be expressed by a variety of immune cells, including natural killer (NK) cells, T cells, NKT, macrophages, and neutrophils [21]. However, their expression differs between Th1 and Th2 cells: TRAIL is observed exclusively in Th2 cells, while FasL and Fas are more abundantly expressed on Th1 cells. Additionally, the higher expression of TRAIL-R1/2 on Th1 increases their susceptibility to apoptosis induced by Th2 cells [21,41,42]. All these membrane-bound ligands and receptors may be released as soluble molecules by proteolytical cleavage. Among them, only soluble FasL was previously reported to increase in the tear fluid collected from ocular cGVHD patients [43]. The chronic inflammation driven by FasL/Fas-mediated apoptosis is involved in conjunctival scarring, meibomian gland dysfunction, and corneal damage, leading to severe dry eye and potential vision loss [44]. In our tear analysis of an acute case of oGVHD, TRAIL-R2 decreased over time, while soluble TRAIL increased, with Fas levels remaining consistently elevated throughout the treatment period (mean value of 963 pg/mL, compared to control tear samples with a mean of 2.69 pg/mL). The chronic oGVHD patient also showed similarly increased Fas levels: 703 pg/mL for the RE and 1080 pg/mL for the LE.

Thus, the other two main groups of soluble markers (those increasing over time or remaining unchanged) suggest a shift from the initial Th1 responses to Th2 and/or Th17 immune responses in the acute case of oGVHD. Apart from TRAIL and Fas, these two last groups also comprised the following soluble molecules discussed below.

The pro-inflammatory chemokines CCL2 and CXCL8/IL-8 were previously shown to be upregulated in the tears of oGVHD patients [43,45]. In our acute oGVHD case, both CCL2 and CXCL8 were initially expressed at significantly high levels, which dropped close to the normal range after three weeks of topical corticosteroid treatment, similarly to the pro-inflammatory cytokines IL-1β, TNF-α, and IL-6. Conversely, in the chronic oGVHD case, both CCL2 and CXCL8 were elevated, along with IL-1β and IL-6, measuring 3–5 times above the normal range. Initially, high levels of these cytokines contribute to the degradation of the ocular surface and the reduction in tear production, exacerbating the dry eye symptoms in oGVHD patients [45,46]. Tear CXCL8 levels were shown by Cocho et al. to be elevated in ocular cGVHD and to positively correlate with conjunctival hyperemia and fluorescein corneal staining, while negatively correlating with Schirmer test scores and TBUT [47]. In our cases, no such clear associations between CXCL8 levels and Schirmer test scores or TBUT were noticed. Interestingly, CXCL5 did not mirror the pattern of CXCL8, but instead aligned with the pattern of IL-17, suggesting the involvement of the CXCL5-Th17/IL-17 axis. This axis has been observed in other chronic ocular surface disorders, and enhances the recruitment of destructive neutrophils, contributing to tissue damage [48,49].

#### 3.2.2. The Second Group of Tear Biomarkers

This group included the anti-inflammatory chemokines and cytokines (CCL5/RANTES, IL-1Ra, IL-4, and IL-10) that showed an increasing trend over time. In our patient with chronic oGVHD, CCL5 levels were 2.5-fold higher than in the healthy control group. Choi W et al. found increased levels of CCL3, CCL4, and CCL5 in the tear film and ocular surface of patients with dry eye syndrome [50]. Pietraszkiewicz et al. found lower CCL5 levels in patients with oGVHD compared to healthy controls, while the study by Cocho et al. did not identify any significant changes of this biomarker’s value in patients with ocular cGVHD compared to a control group of patients with DED of other etiologies [47,51]. The other two chemokines, CCL3 and CCL4, followed a similar trend as CCL5, however, with differences between the two eyes, with higher initial values in the RE compared to the LE. A study performed by Li et al. also identified that these biomarkers were increased in the tears of oGVHD patients compared to healthy controls [45]. IL-1Ra and IL-4 showed a similar pattern to CCL5. They have been previously shown to suppress acute GVHD and play a significant role in the immunoregulation and pathogenesis of allograft-generated effects [52].

The anti-inflammatory cytokine IL-10 exhibited a noticeable difference in the initial values between the two eyes, correlating with the severity of dry eye symptoms (the RE, which presented with more pronounced dry eye symptoms, showed higher initial IL-10 levels). IL-10 is a crucial cytokine in maintaining immunological tolerance, and its deficiency or the absence of its receptor has been implicated in the immunopathology of GVHD [53]. Clinical studies have reported elevated levels of IL-10 and IL-17 on the ocular surface in patients with chronic oGVHD, highlighting their potential role in the disease’s pathogenesis [22,35,36]. IL-10 levels correlated very well with the G-CSF levels. G-CSF is commonly used to boost neutrophil recovery following stem cell transplants. However, its role in the context of GVHD remains controversial. Some studies suggest that G-CSF may help prevent acute GVHD by modulating immune cell profiles, while others have linked its use to an increased risk of developing chronic GVHD. This dual effect highlights the complexity of its influence on the immune system [39,40]. G-CSF, along with GM-CSF and FGF, were also found by Shen et al. to be increased in the tears of ocular cGVHD compared to a control group of DED of other etiologies [43]. In our presented acute oGVHD patient, FGF increased in a pattern similar to IL-4 and CCL5, eventually surpassing the normal range, while the chronic case presented FGF values within the normal limits. FGF is known to play an important role in the regeneration and maintenance of lacrimal gland function, which is often compromised in GVHD. FGF can stimulate the regeneration of epithelial cells in the lacrimal glands and help maintaining a stable tear film. Furthermore, FGF has been suggested to exhibit anti-inflammatory properties, which could mitigate ocular surface inflammation and support overall eye health. FGF administration may even help alleviate the dry eye symptoms and protect lacrimal glands in GVHD, although further research is needed to better understand its efficacy and safety in specific treatments for GVHD patients [54].

#### 3.2.3. The Third Group of Tear Biomarkers

Since GVHD is also associated with the activation of the complement system [25,55], we also monitored the tear levels of the C5a and Factor D complement components and identified high levels in our acute case throughout the observation period. These molecules were thus included in the third group of biomarkers, those that remain consistently elevated or decreased. Interestingly, the investigated chronic patient had only their Factor D levels increased, while their C5a levels were below or within the normal limits. Qiu et al. also studied the role of C5a in acute GVHD and concluded that its level was significantly higher compared to the control group [56]. Additionally, the highly inflammatory environment caused by GVHD can suppress VEGF expression while promoting other inflammatory mediators that influence angiogenesis and tissue repair [57]. VEGF may increase locally in inflamed or damaged tissues, and its levels in tears can differ due to the nature of tear collection methods, disease severity, or degree of lacrimal gland impairment. Additional studies have found inconsistent VEGF activity in GVHD, with some cases showing lower levels of VEGF due to altered immune responses and endothelial activity [58], similar to our results. Importantly, chronic GVHD is associated with fibrosis and damage to the lacrimal glands, reducing their ability to produce not only tears but also essential proteins, including VEGF [59].

### 3.3. Impact of Therapeutic Management on Clinical Outcome and Tear Biomarker Dynamics

The systemic treatments for GVHD help in oGVHD, but the severity of systemic disease cannot be directly correlated with the ocular manifestations [60]. Each therapy line will be discussed further in detail.

Corticosteroids remain the standard first-line therapy in both acute and chronic GVHD, achieving durable responses in 40–50% of patients with cGVHD and less than 50% of patients with aGVHD. Despite continuous research, there are not many randomized controlled trials addressing the treatment of steroid-refractory GVHD (SR-GVHD) [61]. Corticosteroids modulate cytokine production by suppressing pro-inflammatory Th1 and Th17 cytokines (IL-2, IFN-γ, TNF-α, IL-12, IL-6, and IL-17), while promoting anti-inflammatory and immunoregulatory cytokines (IL-10 and TGF-β), leading to a shift from a Th1/Th17-driven response to a more Th2-biased state [62].

Tacrolimus also plays a pivotal role in managing GVHD due to its profound effects on cytokine modulation and T-helper cell responses [63]. Tacrolimus is a calcineurin inhibitor that suppresses T cell activation and proliferation, critical mediators in the pathogenesis of GVHD. Tacrolimus inhibits the production of pro-inflammatory cytokines, such as IL-2, IL-12, IFN-γ, TNF-α, and IL-6, by preventing the activation of NFAT (nuclear factor of activated T cells) [64]. Additionally, it inhibits T cell proliferation and Th1 responses, promoting a shift towards regulatory T cell dominance, boosting IL-10 and TGF-β, both of which have immunosuppressive effects [63]. This mechanism reduces T cell-mediated damage to ocular tissues, including the conjunctiva, cornea, and lacrimal glands, leading to decreased inflammation, stabilization of the tear film, and improving tear production in oGVHD [65,66]. Systemic tacrolimus has been reported to be beneficial for ocular GVHD; however, there are potential adverse reactions to be aware of for long-term systemic administration [67].

Ruxolitinib is a Janus kinase (JAK) 1/2 inhibitor that blocks the signaling of pro-inflammatory cytokines involved in GVHD [68]. A retrospective multicenter study conducted by Zeiser et al. evaluated the efficacy of Ruxolitinib in 54 patients with steroid-refractory acute GVHD (SR-aGVHD), reporting an overall response rate (ORR) of 81.5%, with 46.3% achieving a complete response [69]. Additionally, the phase 2 REACH1 trial, which assessed Ruxolitinib in SR-aGVHD, demonstrated an ORR of 54.9% at day 28, with an overall survival rate of 51% at six months [70,71]. Furthermore, findings from the REACH2 and REACH3 trials indicated that Ruxolitinib significantly prolonged failure-free survival and achieved high response rates in patients with corticosteroid-refractory acute GVHD [69]. Ruxolitinib inhibits cytokine signaling pathways (e.g., IL-6, IL-2, and IFN-γ) mediated by the JAK-STAT pathway, thereby reducing immune overactivation. This action prevents the recruitment and activation of effector T cells responsible for tissue damage [72]. By reducing systemic levels of inflammatory cytokines, Ruxolitinib decreases inflammation and mitigates fibrotic processes in the lacrimal glands, conjunctiva, and cornea [68]. Compared to the best available therapy, Ruxolitinib demonstrated superior response rates and symptom improvement in patients with baseline oGVHD [73]. Clinical studies also suggest that Ruxolitinib offers significant symptomatic relief for patients with oGVHD. Patients experienced a clinically meaningful reduction in the Ocular Surface Disease Index (OSDI) score, with a statistically significant improvement. In most cases, corneal staining was improved, as demonstrated by Seema et al., although there were no significant changes in Schirmer’s test results or oGVHD scores [74].

#### 3.3.1. Impact on Clinical Outcome

In our studied acute case of oGVHD, the marked inflammatory response observed at T0 associated with an OSDI score of 70 might be attributed to an insufficient therapeutic response to systemic corticosteroid treatment. The improvement at T1 to a moderate OSDI score of 30 could be indicative of the effectiveness of the early systemic treatments in controlling inflammation and restoring some degree of ocular function, despite the continuing systemic GVHD. The subsequent reduction in inflammatory markers and improved tear film stability noted at T1 may be due to the introduction of the immunomodulatory agents Tacrolimus and Ruxolitinib. Notably, studies have demonstrated a reduction in corneal staining in patients treated with Ruxolitinib, despite no significant improvement in tear film quantity [74]. This improvement aligns with the clinical expectations in many patients with acute oGVHD, where the reduction of inflammation in the eye can occur even without complete resolution of other systemic symptoms [75]. However, the reappearance of symptoms at T2, marked by the development of mild corneal staining (grade 2 according to Oxford grading), shows how the underlying ocular inflammation can fluctuate, particularly in the context of immunosuppressive therapy and potential treatment resistance in cases of GVHD flare-ups. The reduced tear production and tear film instability observed in the right eye create a vicious cycle where immune dysregulation, inflammation, and fibrosis perpetuate the ocular surface damage. High levels of cytokines such as IL-1β, TNF-α, and IL-6, as well as chemokines like CCL2 and CXCL8, degrade the ocular surface, reduce tear production, and worsen tear film instability. These findings highlight the importance of addressing both inflammatory control and tear film stability in managing ocular GVHD.

#### 3.3.2. Impact on Tear Biomarkers

While the exact effect of systemic therapy on serum levels of other investigated biomarkers, such as CD27, Fas, and the TRAIL/TRAIL-R2 system, remains unknown, it cannot be ruled out that potential changes in the bloodstream might influence their levels in tears in the context of oGVHD. Given the dynamic exchange between systemic circulation and ocular tissues through blood–tear barrier mechanisms, fluctuations in serum concentrations of these biomarkers could have a secondary impact on their presence in tear fluid. In conclusion, for biomarkers such as CD27, TRAIL, and Fas, it remains uncertain whether changes in their tear fluid levels actively contribute to local ocular pathology or merely reflect systemic inflammation. Larger, mechanistic studies incorporating both systemic and local analyses are needed to establish causal relationships, helping to clarify whether these biomarkers are drivers of disease or simply indicators of underlying inflammation.

Overall, our data might also indicate that ocular GVHD can be a dynamic condition in certain patients, where the ocular surface involvement may worsen intermittently despite initial improvement, highlighting the importance of addressing both inflammatory control and tear film stability for continuous monitoring and adaptation of treatment strategies.

## 4. Materials and Methods

### 4.1. Human Study

This study was approved by the institutional ethics committee (Cai Ferate Clinical Hospital of Iasi, 18827/1 October 2024) and informed written consent was obtained from all participants in this study. Both patients (one with acute GVHD and one with chronic GVHD) and healthy volunteers provided both written and verbal permission for their clinical and paraclinical data to be published anonymously. All clinical evaluations were performed by the same physician (M.M.T.Z.) to minimize any potential bias in clinical assessment.

The control group comprised 13 healthy volunteers who fulfilled the following inclusion criteria: (1) absence of ocular surface-related symptoms (OSDI score under 12 points), and (2) Schirmer test without anesthesia > 10 mm in 5 min. The exclusion criteria included the following: (1) previous history of ophthalmic or systemic inflammatory disease, (2) ocular allergy, and (3) current contact lens.

The ophthalmologic assessment included evaluation of the anterior segment and the measurement of various parameters, such as the tear breakup time (TBUT), Schirmer I test, and corneal staining score (Oxford grading). In the acute GVHD patient, anterior segment examination was performed using a portable biomicroscope (Smart Eye Camera, OUI Inc., Tokyo, Japan) due to the patient’s bedridden condition, whereas in the remaining cases, a standard slit-lamp biomicroscope was employed. In addition to the clinical examination, ocular surface symptoms were assessed using the Ocular Surface Disease Index (OSDI). The OSDI is a widely used questionnaire in ophthalmology designed to evaluate the severity of dry eye disease (DED) and its impact on a patient’s quality of life. It consists of 12 questions covering three main domains: ocular symptoms (such as dryness, grittiness, discomfort, painful eyes, and blurred or poor vision), vision-related function (such as difficulty reading, driving at night, or using digital screens, including computers, bank machines, or TVs), and environmental triggers (such as wind, low humidity, or air conditioning). The total OSDI score ranges from 0 to 100, with higher scores indicating more severe symptoms.

### 4.2. Tear Sample Processing

Tear samples were obtained using the Schirmer’s type I tear test (Haag-Streit UK Ltd., Harlow, Essex, UK) without local anesthesia. More precisely, Schirmer strips were placed in the lower eyelid of each eye for five minutes or until fully wetted. Following collection, the strip for each eye was transferred to a separate 2 mL sterile microcentrifuge tube and frozen at −80 °C until further independent analysis.

The Schirmer tear films were defrosted and incubated with 400 μL of 1.5 M Tris–HCl pH 8.8 and protease inhibitors (#A32963 from Thermo Scientific, Waltham, MA, USA) on an orbital shaker (450 rpm) for 3 h at room temperature. After centrifugation at 16,000× *g* for 15 min at 4 °C, the supernatant was collected and the protein concentration was measured using the Nanodrop system (Thermo Fisher Scientific). Next, the concentrations of various cytokines and chemokines from the collected samples were measured using the human custom premixed kits from R&D Systems (Minneapolis, MN, USA) on a Luminex 100/200 platform. Each eye data’s experimental analysis was handled independently.

### 4.3. Tear Cytokine Analysis

The tear biomarkers were assessed using two Luminex multiplex assays performed in parallel: (i) the first one was a Luminex Discovery custom-mix assay including seven biomarkers (beta2-microglobulin (β2-MG), C5a, CD27, Factor D, Fas, TNF-related apoptosis-inducing ligand (TRAIL), and soluble TRAIL receptor 2 (TRAIL-R2)), and (ii) the second one was a Luminex High Performance custom-mix assay comprising 20 other biomarkers: pro-inflammatory and anti-inflammatory chemokines (monocyte chemoattractant protein-1 (MCP-1/CCL2), CCL3/MIP-1α, CCL4/MIP-1β, CCL5/RANTES, CXCL5/ENA-78, and CXCL8/IL-8), growth factors (FGF, VEGF, G-CSF, and GM-CSF), and pro-inflammatory and anti-inflammatory cytokines (TNF-α, IFN-γ, interleukins IL-1α, IL-1β, IL-1Ra, IL-4, IL-5, IL-6, IL-10, and IL-17). The first kit, based on seven biomarkers, required an initial 2-fold dilution, while the second kit, investigating 20 distinct biomarkers, used undiluted samples. The method was previously presented in [12]. Distinct initial standard cocktails were combined with calibrator diluent according to the manufacturer’s instructions to generate the standard 1, which was further used to produce a 3-fold dilution series, up to 6 distinct standards. The standard 1 values for each analyte investigated within the first assay were as follows: 31,235 pg/mL for β2-MG; 1000 pg/mL for C5a; 41,460 pg/mL for CD27; 234,130 pg/mL for Factor D; 35,440 pg/mL for Fas; 9785 pg/mL for TRAIL; and 4740 pg/mL for TRAIL R2. The standard 1 values for the biomarkers investigated with the second kit were as follows: 2400 pg/mL for MCP-1/CCL2; 13,600 pg/mL for CCL3/MIP-1α; 6200 pg/mL for CCL4/MIP-1β; 2300 pg/mL for CCL5/RANTES; 6550 pg/mL for CXCL5/ENA-78; 2750 pg/mL for CXCL8/IL-8; 5200 pg/mL for FGF; 2450 pg/mL for VEGF; 4250 pg/mL for G-CSF; 2700 pg/mL for GM-CSF; 4150 pg/mL for TNF-α; 2800 pg/mL for IFN-γ; 2100 pg/mL for IL-1α; 2150 pg/mL for IL-1β; 4750 pg/mL for IL-1Ra; 3150 pg/mL for IL-4; 1350 pg/mL for IL-5; 4300 pg/mL for IL-6; 2400 pg/mL for IL-10; and 2600 pg/mL for IL-17.

In short, for each Luminex multiplex assay, 50 μL of the standards and samples were incubated with 50 μL of the microparticle mixture in separate wells of a microplate, for 2 h at room temperature on a horizontal orbital microplate shaker set to 500 rpm. After washing 3 times with 100 μL each on a magnetic stand, 50 μL of diluted biotin antibody cocktail were added and the plate was incubated for 1 h under the same conditions. A second wash was followed by the addition of diluted streptavidin–PE solution for 30 min, and the samples were acquired using the Luminex xPONENT Software v4.3 on the Luminex 100/200 platform within 60 min. The acquired data were analyzed using the MATCHIT! Antibody Software v1.5 from IMMUCOR Lifecodes, with calibration curves fitted using a five-parameter logistic (5-PL) regression model. This method ensures accurate quantification by accommodating the asymmetry and variability in immunoassay data, improving the precision of biomarker concentration estimations. Importantly, the simultaneous experimental analysis of 7 or 20 biomarkers significantly reduced the errors associated with sample handling, as it ensured consistency in the processing of biomarkers within the same sample.

The final values were adjusted to the initial protein concentration evaluated with the Nanodrop system from Thermo Fischer Scientific. More precisely, three separate Nanodrop measurements were taken from each sample to determine the protein concentration, and the average of these values was considered the final result. For the control samples, since the data from the LE and RE showed a high correlation (R^2^ > 0.98), the average of the two samples was used in the subsequent statistical interpretations.

### 4.4. Statistical Analysis

Statistical analysis was performed using Graph Pad Prism v5 (Graph Pad Software, San Diego, CA, USA) and SPSS v25 (IBM SPSS Software, Chicago, IL, USA) for estimating the degree of change in tear biomarkers for each individual oGVHD patient, and not for generalization purposes. The *p* values below 0.05 were considered statistically significant. Figures were created with Graph Pad Prism v5, and data are presented as scatter dots or box and whiskers (with minimum and maximum values) plots. The data on tear biomarkers from control subjects were first checked for both normality and variance using the Kolmogorov–Smirnov test. Since these data passed the normality test, the tear biomarkers were further treated as parametric data, and were further analyzed using the two-way repeated measures ANOVA with Bonferroni Correction (Post-hoc Multiple Comparison test). Pearson’s correlation coefficients (R) were used to assess positive or negative associations between measured soluble molecules in the control subjects and the two oGVHD patients. Absolute R values between 0.4 and 0.59 were considered as moderate, between 0.6 and 0.79 as strong, and between 0.8 and 1.0 as very strong correlation factors. Each linear regression graph shows the best-fit line with the 95% confidence band. The coefficient of determination (R^2^) was used as a goodness-of-fit measure, while the F-test was used to determine the level of significance for each linear regression. The heat map analysis presented in Figure 10 was configured using the OriginPro 2024 software, and only serves to visually illustrate the potential differences observed in the associations between various biomarkers within the acute oGVHD patient over time, as well as the healthy control group. Considering the differences in the progression of DED between the two eyes in the acute oGVHD patient, data for each eye were included independently in the analysis. For the control group, to reduce the bias associated with dependent analysis, we used the average individual data, as previously mentioned.

Since no proper power calculation analysis was performed, the small sample size is a key limitation for our study to provide generalized conclusions for disease. The reported changes are patient-specific.

## 5. Conclusions

Being limited to only two cases of ocular GVHD, our pilot study offers preliminary, hypothesis-generating findings on the potential use of novel biomarkers that may warrant further extensive investigation in larger cohorts. Overall, among the newly proposed biomarkers that showed differences between the two oGVHD patients, and the limited control group, we could mention the soluble forms of CD27, Fas, TRAIL-R2, and TRAIL. While CD27 and Fas were initially elevated in both oGVHD patients (molecules which were undetectable in control subjects), TRAIL-R2 was only detectable in the acute oGVHD case (and not in the chronic GVHD patient, nor in the investigated control subjects), with TRAIL being initially reduced in the acute oGVHD case when compared to the chronic oGVHD case or controls. This study, although limited to two patients with oGVHD, might raise the hypothesis that potential differences in tear biomarkers might exist between acute and chronic oGVHD, which should be carefully monitored throughout the treatment period to better understand disease progression and refine management strategies.

### Future Directions

Our observations are particularized to only two oGVHD subjects, being only a pilot study. Thus, we encourage larger prospective studies and rigorous clinical trials to systematically investigate the dynamics of the presented biomarkers through repeated tear collections from both acute and chronic oGVHD patients, while controlling for confounding factors such as the administered systemic therapy, and performing formal power analyses. Several other questions also emerged from out pilot analysis. It still remains unclear if the observed changes in tear fluid biomarker levels drive the local pathology or they simply mirror systemic inflammation. To clarify this cause-and-effect relationship, larger mechanistic studies are required where both tear and serum/plasma fluids should be simultaneously investigated. Additionally, further studies are also needed to investigate whether the proposed biomarkers are specific to oGVHD or are common to other conditions associated with dry eye symptoms.

Such studies could help clarify the potential correlation between disease severity, stages, and tear cytokine levels, as well as address open questions regarding the most effective therapeutic strategies and the feasibility of using these biomarkers to predict disease progression in conjunction with specific treatment approaches.

## Figures and Tables

**Table 1:** Tear levels of biomarkers in control subjects and two cases of acute or chronic ocular GVHD.

Variable	aGVHD RE T0	aGVHD LE T0	aGVHD RE T1	aGVHD LE T1	aGVHD RE T2	aGVHD LE T2	cGVHD RE T0	cGVHD LE T0	Controls Mean ± S.E.M.
Schirmer I test (mm)	30	25	8	7	5	5	12	7	24.15 ± 2.68
Proteins (mg/mL)	2.59	2.95	1.15	0.93	0.77	0.92	1.03	1.01	1.17 ± 0.09
β2-MG (μg/mL)	103.89	58.71	227.88	230.27	190.05	122.98	307.86	284.64	258.52 ± 15.7
C5a (pg/mL)	46.52	30.81	32.07	45.59	65.09	24.1	5.9	20.14	16.86 ± 1.17
CD27 (pg/mL)	297.79	288.13	0	0.79	0	20.29	71.05	125.89	0.93 ± 0.93
Factor D (μg/mL)	13.36	12.01	4.5	4.64	10.01	17.53	9	13.17	3.1 ± 0.36
Fas (pg/mL)	956.98	963.82	640.23	562.92	1337.4	1316.85	703.62	1080.14	2.69 ± 2.69
TRAIL-R2 (pg/mL)	95.12	99.99	13.29	16.52	0	0	0	0	0 ± 0
TRAIL (pg/mL)	79.83	25.26	166.37	73.81	146.33	216.89	642.33	470.32	1003.19 ± 186.47
CCL2 (pg/mL)	658.07	564	48.4	70.99	9.33	10.86	58.45	30.08	14.17 ± 2.99
CCL3 (pg/mL)	291.65	115.29	447.32	497.2	646.18	563.95	559.68	541.16	423.23 ± 25.94
CCL4 (pg/mL)	42.22	20.61	47.94	50.42	70.45	59.97	51.2	55	45.35 ± 2.44
CCL5 (pg/mL)	15.03	9.56	31.62	35.13	44.66	47.51	88.3	84.41	31.49 ± 2.51
CXCL5 (pg/mL)	58.08	46.56	174.39	224.25	127.25	123.98	299.65	189.8	82.48 ± 5
CXCL8 (pg/mL)	728.86	760.84	195.89	193.93	143.5	154.51	457.47	539.93	60.3 ± 17.7
FGF (pg/mL)	76.84	103.61	228.11	234.36	329.55	301.72	227.17	240.08	199.62 ± 12.58
G-CSF (pg/mL)	103.4	1.36	23.1	27.08	28.57	22.14	136.44	148.74	16.13 ± 2.63
GM-CSF (pg/mL)	7.34	4.94	24.78	24.87	37.01	31.58	26.47	23.51	19.49 ± 1.31
IFN-γ (pg/mL)	2.35	1.26	12.28	11.19	16.81	15.19	11.2	12.3	10.29 ± 0.66
IL-1α (pg/mL)	70.49	58.41	56.54	61.21	79.75	65.49	57.46	60.06	49.77 ± 2.88
IL-1β (pg/mL)	736.01	777.18	16.4	35.12	15.29	14.01	65.9	116.62	7.74 ± 1.89
IL-1Ra (pg/mL)	9.27	6.6	67.13	72.67	119.92	92.34	57.77	63.48	55.95 ± 4.59
IL-4 (pg/mL)	34.88	25.22	86.58	89.58	132.83	109.2	82.27	89.24	75.35 ± 4.8
IL-5 (pg/mL)	0.26	0.25	0.17	0.16	0.19	0.16	0.004	0.21	0.07 ± 0.11
IL-6 (pg/mL)	49.76	18.26	8.24	8.46	9.31	8.11	14.2	11.41	4.61 ± 0.26
IL-10 (pg/mL)	108.96	12.33	61.17	63.69	76.52	76.12	72.38	73.59	43.97 ± 2.92
IL-17 (pg/mL)	0.04	0.01	3.42	3.51	6.89	15.14	3.06	2.53	1.74 ± 0.23
TNF-α(pg/mL)	28.65	21.29	11.73	12.48	17.77	15.43	13.26	14.15	10.37 ± 0.66
VEGF (pg/mL)	53.55	28.81	40.8	32.96	24.19	15.76	65.11	49.07	112.81 ± 9.56

Abbreviations: GVHD = graft versus host disease; RE = right eye; LE = left eye; S.E.M. = standard error of the mean.

## Data Availability

The original contributions presented in the study are included in the article. Further inquiries can be directed to the corresponding author.

## Abbreviations

The following abbreviations are used in this manuscript:

oGVHD	Ocular graft versus host disease
cGVHD	Chronic graft versus host disease
RE	Right eye
LE	Left eye
β2-MG	beta2-microglobulin
TRAIL	TNF-related apoptosis-inducing ligand
TRAIL-R2	TRAIL receptor 2
CCL2/MCP-1	Chemokine ligand 2/Monocyte chemoattractant protein-1
CCL3/MIP-1α	Chemokine ligand 3/Macrophage inflammatory protein 1-alpha
CCL4/MIP-1β	Chemokine ligand 4/Macrophage inflammatory protein 1-beta
CCL5/RANTES	Chemokine ligand 5/Regulated upon Activation, Normal T cell Expressed and presumably Secreted
CXCL5/ENA-78	C-X-C motif chemokine 5/Epithelial cell-derived neutrophil-activating peptide
CXCL8/IL-8	C-X-C motif chemokine 5/Interleukin 8
FGF	Fibroblast growth factor
VEGF	Vascular endothelial growth factor
G-CSF	Granulocyte colony-stimulating factor
GM-CSF	Granulocyte-macrophage colony-stimulating factor
TNF-α	Tumor necrosis factor-alpha
IFN-γ	Interferon gamma
IL-1Ra	Interleukin-1 receptor antagonist
sem	Standard error of the mean

## References

[1] Gratwohl A., Pasquini M.C., Aljurf M., Atsuta Y., Baldomero H., Foeken L., Gratwohl M., Bouzas L.F., Confer D., Frauendorfer K. (2015). One Million Haemopoietic Stem-Cell Transplants: A Retrospective Observational Study. Lancet Haematol..

[2] Justiz Vaillant A.A., Modi P., Mohammadi O. (2024). Graft-Versus-Host Disease.

[3] Carreno-Galeano J.T., Dohlman T.H., Kim S., Yin J., Dana R. (2021). A Review of Ocular Graft-versus-Host Disease: Pathophysiology, Clinical Presentation and Management. Ocul. Immunol. Inflamm..

[4] Singh R.B., Cho W., Liu C., Naderi A., Surico P.L., Kahale F., Dohlman T.H., Chauhan S.K., Dana R. (2024). Immunopathological Mechanisms and Clinical Manifestations of Ocular Graft-versus-Host Disease Following Hematopoietic Stem Cell Transplantation. Bone Marrow Transplant..

[5] Ban Y., Ogawa Y., Ibrahim O.M.A., Tatematsu Y., Kamoi M., Uchino M., Yaguchi S., Dogru M., Tsubota K. (2011). Morphologic Evaluation of Meibomian Glands in Chronic Graft-versus-Host Disease Using in Vivo Laser Confocal Microscopy. Mol. Vis..

[6] Inamoto Y., Valdés-Sanz N., Ogawa Y., Alves M., Berchicci L., Galvin J., Greinix H., Hale G.A., Horn B., Kelly D. (2019). Ocular Graft-versus-Host Disease after Hematopoietic Cell Transplantation: Expert Review from the Late Effects and Quality of Life Working Committee of the CIBMTR and Transplant Complications Working Party of the EBMT. Bone Marrow Transplant..

[7] Cheng X., Huang R., Huang S., Fan W., Yuan R., Wang X., Zhang X. (2023). Recent Advances in Ocular Graft-versus-Host Disease. Front. Immunol..

[8] Filipovich A.H., Weisdorf D., Pavletic S., Socie G., Wingard J.R., Lee S.J., Martin P., Chien J., Przepiorka D., Couriel D. (2005). National Institutes of Health Consensus Development Project on Criteria for Clinical Trials in Chronic Graft-versus-Host Disease: I. Diagnosis and Staging Working Group Report. Biol. Blood Marrow Transplant. J. Am. Soc. Blood Marrow Transplant..

[9] Pellegrini M., Bernabei F., Barbato F., Arpinati M., Giannaccare G., Versura P., Bonifazi F. (2021). Incidence, Risk Factors and Complications of Ocular Graft-Versus-Host Disease Following Hematopoietic Stem Cell Transplantation. Am. J. Ophthalmol..

[10] Craig J.P., Nichols K.K., Akpek E.K., Caffery B., Dua H.S., Joo C.-K., Liu Z., Nelson J.D., Nichols J.J., Tsubota K. (2017). TFOS DEWS II Definition and Classification Report. Ocul. Surf..

[11] Tamhane M., Cabrera-Ghayouri S., Abelian G., Viswanath V. (2019). Review of Biomarkers in Ocular Matrices: Challenges and Opportunities. Pharm. Res..

[12] Pavel-Tanasa M., Constantinescu D., Cianga C.M., Anisie E., Mereuta A.I., Tuchilus C.G., Cianga P. (2022). Adipokines, and Not Vitamin D, Associate with Antibody Immune Responses Following Dual BNT162b2 Vaccination within Individuals Younger than 60 Years. Front. Immunol..

[13] Saleki K., Shirzad M., Javanian M., Mohammadkhani S., Alijani M.H., Miri N., Oladnabi M., Azadmehr A. (2022). Serum Soluble Fas Ligand Is a Severity and Mortality Prognostic Marker for COVID-19 Patients. Front. Immunol..

[14] Yamada A., Arakaki R., Saito M., Kudo Y., Ishimaru N. (2017). Dual Role of Fas/FasL-Mediated Signal in Peripheral Immune Tolerance. Front. Immunol..

[15] Matsumoto H., Murakami Y., Kataoka K., Notomi S., Mantopoulos D., Trichonas G., Miller J.W., Gregory M.S., Ksander B.R., Marshak-Rothstein A. (2015). Membrane-Bound and Soluble Fas Ligands Have Opposite Functions in Photoreceptor Cell Death Following Separation from the Retinal Pigment Epithelium. Cell Death Dis..

[16] Gonçalves I., Singh P., Tengryd C., Cavalera M., Yao Mattisson I., Nitulescu M., Flor Persson A., Volkov P., Engström G., Orho-Melander M. (2019). STRAIL-R2 (Soluble TNF [Tumor Necrosis Factor]-Related Apoptosis-Inducing Ligand Receptor 2) a Marker of Plaque Cell Apoptosis and Cardiovascular Events. Stroke.

[17] Staniek J., Lorenzetti R., Heller B., Janowska I., Schneider P., Unger S., Warnatz K., Seidl M., Venhoff N., Thiel J. (2019). TRAIL-R1 and TRAIL-R2 Mediate TRAIL-Dependent Apoptosis in Activated Primary Human B Lymphocytes. Front. Immunol..

[18] Naval J., de Miguel D., Gallego-Lleyda A., Anel A., Martinez-Lostao L. (2019). Importance of TRAIL Molecular Anatomy in Receptor Oligomerization and Signaling. Implications for Cancer Therapy. Cancers.

[19] Fenton M.J., Buras J.A., Donnelly R.P. (1992). IL-4 Reciprocally Regulates IL-1 and IL-1 Receptor Antagonist Expression in Human Monocytes. J. Immunol..

[20] Mühl H., Pfeilschifter J. (2003). Anti-Inflammatory Properties of pro-Inflammatory Interferon-Gamma. Int. Immunopharmacol..

[21] Falschlehner C., Schaefer U., Walczak H. (2009). Following TRAIL’s Path in the Immune System. Immunology.

[22] Janjic B.M., Kulkarni A., Ferris R.L., Vujanovic L., Vujanovic N.L. (2022). Human B Cells Mediate Innate Anti-Cancer Cytotoxicity Through Concurrent Engagement of Multiple TNF Superfamily Ligands. Front. Immunol..

[23] Grisanti L.A. (2023). TRAIL and Its Receptors in Cardiac Diseases. Front. Physiol..

[24] Nguyen H., Alawieh A., Bastian D., Kuril S., Dai M., Daenthanasanmak A., Zhang M., Iamsawat S., Schutt S.D., Wu Y. (2020). Targeting the Complement Alternative Pathway Permits Graft Versus Leukemia Activity While Preventing Graft Versus Host Disease. Clin. Cancer Res. Off. J. Am. Assoc. Cancer Res..

[25] Tsakiris D.A., Gavriilaki E., Chanou I., Meyer S.C. (2024). Hemostasis and Complement in Allogeneic Hematopoietic Stem Cell Transplantation: Clinical Significance of Two Interactive Systems. Bone Marrow Transplant..

[26] Speidl W.S., Kastl S.P., Hutter R., Katsaros K.M., Kaun C., Bauriedel G., Maurer G., Huber K., Badimon J.J., Wojta J. (2011). The Complement Component C5a Is Present in Human Coronary Lesions in Vivo and Induces the Expression of MMP-1 and MMP-9 in Human Macrophages in Vitro. FASEB J. Off. Publ. Fed. Am. Soc. Exp. Biol..

[27] Bystry R.S., Aluvihare V., Welch K.A., Kallikourdis M., Betz A.G. (2001). B Cells and Professional APCs Recruit Regulatory T Cells via CCL4. Nat. Immunol..

[28] Reshef R., Luger S.M., Hexner E.O., Loren A.W., Frey N.V., Nasta S.D., Goldstein S.C., Stadtmauer E.A., Smith J., Bailey S. (2012). Blockade of Lymphocyte Chemotaxis in Visceral Graft-versus-Host Disease. N. Engl. J. Med..

[29] Morikawa K., Miyawaki T., Oseko F., Morikawa S., Imai K. (1993). G-CSF Enhances the Immunoglobulin Generation Rather than the Proliferation of Human B Lymphocytes. Eur. J. Haematol..

[30] Itoh K., Hirohata S. (1995). The Role of IL-10 in Human B Cell Activation, Proliferation, and Differentiation. J. Immunol..

[31] Lighaam L.C., Unger P.-P.A., Vredevoogd D.W., Verhoeven D., Vermeulen E., Turksma A.W., Ten Brinke A., Rispens T., van Ham S.M. (2018). In Vitro-Induced Human IL-10(+) B Cells Do Not Show a Subset-Defining Marker Signature and Plastically Co-Express IL-10 With Pro-Inflammatory Cytokines. Front. Immunol..

[32] Nova-Lamperti E., Fanelli G., Becker P.D., Chana P., Elgueta R., Dodd P.C., Lord G.M., Lombardi G., Hernandez-Fuentes M.P. (2016). IL-10-Produced by Human Transitional B-Cells down-Regulates CD86 Expression on B-Cells Leading to Inhibition of CD4+T-Cell Responses. Sci. Rep..

[33] Russell J.H., Ley T.J. (2002). Lymphocyte-Mediated Cytotoxicity. Annu. Rev. Immunol..

[34] Du W., Cao X. (2018). Cytotoxic Pathways in Allogeneic Hematopoietic Cell Transplantation. Front. Immunol..

[35] Takada S., Hatsumi N., Saito T., Matsushima T., Sakura T., Tamura J., Karasawa M., Miyawaki S. (2000). Two Cases of Chronic Graft-versus-Host Disease with Elevated Levels of Soluble Fas Ligand in Serum. Am. J. Hematol..

[36] Kim S.K. (2005). Ocular Graft vs. Host Disease. Ocul. Surf..

[37] Masalkhi M., Wahoud N., Moran B., Elhassadi E. (2024). Evolving Therapeutic Paradigms in Ocular Graft-versus-Host Disease. Eye.

[38] Royer D.J., Echegaray-Mendez J., Lin L., Gmyrek G.B., Mathew R., Saban D.R., Perez V.L., Carr D.J. (2019). Complement and CD4(+) T Cells Drive Context-Specific Corneal Sensory Neuropathy. Elife.

[39] Tappeiner C., Heiligenhaus A., Halter J.P., Miserocchi E., Bandello F., Goldblum D. (2023). Challenges and Concepts in the Diagnosis and Management of Ocular Graft-versus-Host Disease. Front. Med..

[40] Paranga T.G., Pavel-Tanasa M., Constantinescu D., Iftimi E., Plesca C.E., Miftode I.-L., Cianga P., Miftode E. (2024). Distinct Soluble Immune Checkpoint Profiles Characterize COVID-19 Severity, Mortality and SARS-CoV-2 Variant Infections. Front. Immunol..

[41] Roberts A.I., Devadas S., Zhang X., Zhang L., Keegan A., Greeneltch K., Solomon J., Wei L., Das J., Sun E. (2003). The Role of Activation-Induced Cell Death in the Differentiation of T-Helper-Cell Subsets. Immunol. Res..

[42] Zhang X.R., Zhang L.Y., Devadas S., Li L., Keegan A.D., Shi Y.F. (2003). Reciprocal Expression of TRAIL and CD95L in Th1 and Th2 Cells: Role of Apoptosis in T Helper Subset Differentiation. Cell Death Differ..

[43] Shen Z., Ma J., Peng R., Hu B., Zhao Y., Liu S., Hong J. (2022). Biomarkers in Ocular Graft-Versus-Host Disease: Implications for the Involvement of B Cells. Transplant. Cell. Ther..

[44] Wu Q., Chen H., Fang J., Xie W., Hong M., Xia L. (2012). Elevated Fas/FasL System and Endothelial Cell Microparticles Are Involved in Endothelial Damage in Acute Graft-versus-Host Disease: A Clinical Analysis. Leuk. Res..

[45] Li A.J., Martinez-Guasch F., Sajjan S., Cao X., Atanackovic D., Sunshine S. (2023). Tear Cytokine Profiling for Ocular Graft versus Host Disease Utilizing a New Multiplex Assay. J. Immunol..

[46] Antin J.H., Weisdorf D., Neuberg D., Nicklow R., Clouthier S., Lee S.J., Alyea E., McGarigle C., Blazar B.R., Sonis S. (2002). Interleukin-1 Blockade Does Not Prevent Acute Graft-versus-Host Disease: Results of a Randomized, Double-Blind, Placebo-Controlled Trial of Interleukin-1 Receptor Antagonist in Allogeneic Bone Marrow Transplantation. Blood.

[47] Cocho L., Fernández I., Calonge M., Martínez V., González-García M.J., Caballero D., López-Corral L., García-Vázquez C., Vázquez L., Stern M.E. (2016). Biomarkers in Ocular Chronic Graft Versus Host Disease: Tear Cytokine- and Chemokine-Based Predictive Model. Investig. Ophthalmol. Vis. Sci..

[48] Fan N.-W., Dohlman T.H., Foulsham W., McSoley M., Singh R.B., Chen Y., Dana R. (2021). The Role of Th17 Immunity in Chronic Ocular Surface Disorders. Ocul. Surf..

[49] Disteldorf E.M., Krebs C.F., Paust H.-J., Turner J.-E., Nouailles G., Tittel A., Meyer-Schwesinger C., Stege G., Brix S., Velden J. (2015). CXCL5 Drives Neutrophil Recruitment in TH17-Mediated GN. J. Am. Soc. Nephrol..

[50] Choi W., Li Z., Oh H.-J., Im S.-K., Lee S.-H., Park S.-H., You I.-C., Yoon K.-C. (2012). Expression of CCR5 and Its Ligands CCL3, -4, and -5 in the Tear Film and Ocular Surface of Patients with Dry Eye Disease. Curr. Eye Res..

[51] Pietraszkiewicz A.A., Payne D., Abraham M., Garced A., Devarasetty K.C., Wall M., Menezes S.M., Ugarte S., Pirsl F., Goklemez S. (2021). Ocular Surface Indicators and Biomarkers in Chronic Ocular Graft-versus-Host Disease: A Prospective Cohort Study. Bone Marrow Transplant..

[52] Via C.S., Soloviova K., Puliaiev M., Puliav R., Puliaeva I., Morris S.C., Finkelman F.D. (2017). In Vivo IL-4 Prevents Allo-Antigen Driven CD8+ CTL Development. Clin. Immunol..

[53] Zhang P., Hill G.R. (2019). Interleukin-10 Mediated Immune Regulation after Stem Cell Transplantation: Mechanisms and Implications for Therapeutic Intervention. Semin. Immunol..

[54] Panoskaltsis-Mortari A., Lacey D.L., Vallera D.A., Blazar B.R. (1998). Keratinocyte Growth Factor Administered Before Conditioning Ameliorates Graft-Versus-Host Disease After Allogeneic Bone Marrow Transplantation in Mice. Blood.

[55] Bohlen J., Gomez C., Zhou J., Martinez Guasch F., Wandvik C., Sunshine S.B. (2024). Molecular Biomarkers in Ocular Graft-versus-Host Disease: A Systematic Review. Biomolecules.

[56] Qiu Y., Hu B., Peng R.-M., Huang J.-F., Hong J. (2022). Tear Cytokines as Biomarkers for Acute Ocular Graft-Versus-Host Disease. Cornea.

[57] de Almeida Borges D., Alborghetti M.R., Franco Paes Leme A., Ramos Domingues R., Duarte B., Veiga M., Trindade Ferrer M., Viana Wanzeler A.C., Leite Arieta C.E., Alves M. (2020). Tear Proteomic Profile in Three Distinct Ocular Surface Diseases: Keratoconus, Pterygium, and Dry Eye Related to Graft-versus-Host Disease. Clin. Proteom..

[58] Riesner K., Shi Y., Jacobi A., Kräter M., Kalupa M., McGearey A., Mertlitz S., Cordes S., Schrezenmeier J.-F., Mengwasser J. (2017). Initiation of Acute Graft-versus-Host Disease by Angiogenesis. Blood.

[59] Penack O., Socié G., van den Brink M.R.M. (2011). The Importance of Neovascularization and Its Inhibition for Allogeneic Hematopoietic Stem Cell Transplantation. Blood.

[60] Shikari H., Antin J.H., Dana R. (2013). Ocular Graft-versus-Host Disease: A Review. Surv. Ophthalmol..

[61] Zhang M.-Y., Zhao P., Zhang Y., Wang J.-S. (2022). Efficacy and Safety of Ruxolitinib for Steroid-Refractory Graft-versus-Host Disease: Systematic Review and Meta-Analysis of Randomised and Non-Randomised Studies. PLoS ONE.

[62] Hill G.R., Koyama M. (2020). Cytokines and Costimulation in Acute Graft-versus-Host Disease. Blood.

[63] Henden A.S., Hill G.R. (2015). Cytokines in Graft-versus-Host Disease. J. Immunol..

[64] Yu Y., Zhong J., Peng L., Wang B., Li S., Huang H., Deng Y., Zhang H., Yang R., Wang C. (2017). Tacrolimus Downregulates Inflammation by Regulating Pro_/Anti_inflammatory Responses in LPS_induced Keratitis. Mol. Med. Rep..

[65] Nash R.A., Antin J.H., Karanes C., Fay J.W., Avalos B.R., Yeager A.M., Przepiorka D., Davies S., Petersen F.B., Bartels P. (2000). Phase 3 Study Comparing Methotrexate and Tacrolimus with Methotrexate and Cyclosporine for Prophylaxis of Acute Graft-versus-Host Disease after Marrow Transplantation from Unrelated Donors. Blood.

[66] Surico P.L., Luo Z.K. (2024). Understanding Ocular Graft-versus-Host Disease to Facilitate an Integrated Multidisciplinary Approach. Transplant. Cell. Ther..

[67] Hessen M., Akpek E.K. (2012). Ocular Graft-versus-Host Disease. Curr. Opin. Allergy Clin. Immunol..

[68] Quintás-Cardama A., Vaddi K., Liu P., Manshouri T., Li J., Scherle P.A., Caulder E., Wen X., Li Y., Waeltz P. (2010). Preclinical Characterization of the Selective JAK1/2 Inhibitor INCB018424: Therapeutic Implications for the Treatment of Myeloproliferative Neoplasms. Blood.

[69] Zeiser R., von Bubnoff N., Butler J., Mohty M., Niederwieser D., Or R., Szer J., Wagner E.M., Zuckerman T., Mahuzier B. (2020). Ruxolitinib for Glucocorticoid-Refractory Acute Graft-versus-Host Disease. N. Engl. J. Med..

[70] Choi J., Cooper M.L., Alahmari B., Ritchey J., Collins L., Holt M., DiPersio J.F. (2014). Pharmacologic Blockade of JAK1/JAK2 Reduces GvHD and Preserves the Graft-versus-Leukemia Effect. PLoS ONE.

[71] Hechinger A.-K., Smith B.A.H., Flynn R., Hanke K., McDonald-Hyman C., Taylor P.A., Pfeifer D., Hackanson B., Leonhardt F., Prinz G. (2015). Therapeutic Activity of Multiple Common γ-Chain Cytokine Inhibition in Acute and Chronic GVHD. Blood.

[72] Yeleswaram S., Smith P., Burn T., Covington M., Juvekar A., Li Y., Squier P., Langmuir P. (2020). Inhibition of Cytokine Signaling by Ruxolitinib and Implications for COVID-19 Treatment. Clin. Immunol..

[73] Sunshine S., Bhatt V., Galvin J., Tian C., Zieser R. (2023). Oral Ruxolitinib Treatment for Patients with Ocular Manifestations of Chronic Graft-versus-Host Disease: A Post Hoc Analysis of the Phase 3 REACH3 Study. Investig. Ophthalmol. Vis. Sci..

[74] Sajjan S., Tibbs E., Utz M., Rapoport A.P., Yared J., Dahiya S., Cao X., Hardy N., Sunshine S.B. (2023). Can Janus Kinase Inhibition Improve Ocular Graft versus Host Disease?. Ocul. Surf..

[75] Nassar A., Tabbara K.F., Aljurf M. (2013). Ocular Manifestations of Graft-versus-Host Disease. Saudi J. Ophthalmol. Off. J. Saudi Ophthalmol. Soc..

